# Do Scientific Advancements Lean on the Shoulders of Giants? A Bibliometric Investigation of the Ortega Hypothesis

**DOI:** 10.1371/journal.pone.0013327

**Published:** 2010-10-13

**Authors:** Lutz Bornmann, Félix de Moya Anegón, Loet Leydesdorff

**Affiliations:** 1 Office of Research Analysis and Foresight, Max Planck Society, Munich, Germany; 2 CSIC/CCHS/IPP, SCImago Group (Spain), Communication and Information Science Faculty, University of Granada, Granada, Spain; 3 Amsterdam School of Communication Research (ASCoR), University of Amsterdam, Amsterdam, The Netherlands; Cuban Neuroscience Center, Cuba

## Abstract

**Background:**

In contrast to Newton's well-known aphorism that he had been able “to see further only by standing on the shoulders of giants,” one attributes to the Spanish philosopher Ortega y Gasset the hypothesis saying that top-level research cannot be successful without a mass of medium researchers on which the top rests comparable to an iceberg.

**Methodology/Principal Findings:**

The Ortega hypothesis predicts that highly-cited papers and medium-cited (or lowly-cited) papers would equally refer to papers with a medium impact. The Newton hypothesis would be supported if the top-level research more frequently cites previously highly-cited work than that medium-level research cites highly-cited work. Our analysis is based on (i) all articles and proceedings papers which were published in 2003 in the life sciences, health sciences, physical sciences, and social sciences, and (ii) all articles and proceeding papers which were cited within these publications. The results show that highly-cited work in all scientific fields more frequently cites previously highly-cited papers than that medium-cited work cites highly-cited work.

**Conclusions/Significance:**

We demonstrate that papers contributing to the scientific progress in a field lean to a larger extent on previously important contributions than papers contributing little. These findings support the Newton hypothesis and call into question the Ortega hypothesis (given our usage of citation counts as a proxy for impact).

## Introduction

“La ciencia experimental ha progresado en buena parte merced al trabajo de hombres fabulosamente mediocres, y aun menos que mediocres” Ortega y Gasset

In contrast to Newton's [1], [2] well-known aphorism that he had been able “to see further only by standing on the shoulders of giants,” one attributes to the Spanish philosopher Ortega y Gasset the hypothesis saying that top-level research cannot be successful without a mass of medium researchers on which the top rests comparable to an iceberg [3], [4]. A third possibility offered by Turner and Chubin [5] is the so-called Ecclesiastes hypothesis: these authors argue that scientific advancements can be considered as the result of chance processes or fortune using an evolutionary model of science. The issue, discussed by many eminent scientists and philosophers, is highly relevant for today's research funding policies. From this perspective, one can discuss whether research funding should be focused on elite scientists or rather aim at generating scientific capacities in the broad range of scientists.

In this study, we address this question from a bibliometric perspective using capabilities in literature databases that became recently available [6]: Unlike the (Social) Science Citation Index of Thomson Reuters, the Scopus database of Elsevier—launched in 2004—enables us to determine whether highly-cited papers themselves cite highly-cited papers to a significant extent. This provides some insights into whether giants in research like to build on the research of other giants. We gained these insights into four major fields of science: physical sciences, life sciences, health sciences, and social sciences. Both the citing and the cited papers were identified within the field-specific journal sets covered by the Scopus database. From a sociological perspective, our bibliometric approach may have only limited value because citations are an imperfect proxy for the actual usage of research results. Citations are just one parameter of scientific quality. However, the strength of this approach lies in the large number of observations that can be evaluated. Statistical analyses of bibliometric data may allow us to cast new light on the validity of the three hypotheses and give insights into the expected effects of different research funding models.

Recently, there is a trend away from a model to allocate research funds on the basis of block grants to institutions towards resource allocation based on the principle of merit of individual researchers [7]. Institutional allocation which follows a principle of equality (everyone gets an equal share) can perhaps be legitimated in terms of the Ortega hypothesis more than in terms of the elite structure proposed by the Newton hypothesis [8]. In the latter model funding were to be concentrated to the top scientists in order to create a critical mass of elite scientists [9]. This focus on top quality can perhaps be justified by the wish to obtain increased accountability of academic research [10].

Although we witness an increased focus on excellence in science funding [11], it is yet unclear which of the three competing hypotheses can be supported by the data. Is top-level research systematically connected to top-level research in the past or does top-level research also presume research at the medium level? The few studies which study the empirical merit of the three hypotheses were mostly published several years ago and based on small samples within single disciplines [12].

## Methods

Using citation analysis for the operationalisation, two basic assumptions are made. From the citing perspective, one assumes that papers cited by scientists represent a roughly valid indicator of influence on their work [13]. A cited reference can perhaps be considered as a reward for the usefulness of the cited paper [14]. The aggregate of cited references in a paper can be considered as indicating the theoretical and empirical resources for building an author's argument [15]. However, individual papers may accumulate citations for trivial reasons [16]. In the case of large numbers, these deviances may be averaged out. Thus, with sufficiently large numbers (e.g., a group of researchers as a whole over a longer period of time) citation frequency can be assumed as a proxy for impact [17].

Our study is based on the Scopus database that offers the possibility of direct coupling between the cited references within a paper and their respective numbers of citations. In the (Social) Science Citation Index, a two-steps coupling procedure is then needed and the procedure is error-prone because the information in the cited references is often incomplete. In Scopus, cited references are uniquely identified as previously published papers. Although the Scopus database indexes more journals than Thomson Reuters' citation indexes [18], it may also contain more peripheral journals, that is, journals publishing papers with low visibility or publishing papers without applying the peer review process [19]. In order to control for this effect we use the intersection of 6,578 journals between the Scopus set (n = 17,087) and the (Social) Science Citation Index set (n = 7,612) in the publication year 2003. This group of journals is acknowledged by the teams at both Thomson Reuters and Elsevier as of sufficient visibility to warrant inclusion into its set. In other words, to use only journals in our study with a “higher” visibility (quality), we restricted the Scopus journal set to those journals that are also included in the (Social) Science Citation Index.

Like in the citation indexes of Thomson Reuters, scientific fields are defined in Scopus in terms of journal sets. There are 305 “specific subject areas” (e.g., “Biochemistry”) organized into 26 “subject areas” (e.g., “Biochemistry, Genetics & Molecular Biology”), plus a “general subject area” containing multidisciplinary journals such as *Nature* or *Science* [20]. The subject areas (with the exception of the “general subject area”) are grouped into four main fields:

1. Life Sciences: Agricultural & Biological Sciences; Biochemistry, Genetics & Molecular Biology; Immunology & Microbiology; Neuroscience; Pharmacology, Toxicology & Pharmaceutics.
2. Health Sciences: Medicine; Nursing; Veterinary; Dentistry; Health Professions.
3. Physical Sciences: Chemical Engineering; Chemistry; Computer Science; Earth & Planetary Science; Energy; Engineering; Environmental Science; Materials Science; Mathematics; Physics & Astronomy.
4. Social Sciences: Arts & Humanities; Business, Management & Accounting; Decision Sciences; Economics, Econometrics and Finance; Psychology; Social Sciences.

Our analysis is based on (*i*) all articles and proceedings papers which were published in 2003 in the life sciences (n = 248,812), health sciences (n = 210,758), physical sciences (n = 366,974), and social sciences (n = 41,095), and (*ii*) all articles and proceeding papers which were cited within these publications. These cited references amount to: life sciences (n = 3,809,845), health sciences (n = 2,373,799), physical sciences (n = 3,317,683), and social sciences (n = 278,146). We only included references to papers published within the Scopus journal set since no citation data is available for papers outside this set. Since researchers grouped in the social sciences category frequently publish in books and non-English journals, the numbers in this area are smaller than in the life sciences, health sciences, and physical sciences [6], [20]. (A similar difference can be found between the Science Citation Index and Social Sciences Citation Index, respectively.) As Scopus provides reliable citation coverage only from 1996 onwards [21], we included only cited references published since that date.

We studied the citation impact of the papers which are cited in all the papers with publication year 2003. As normalizations, first, the citation windows are set to five years after the year of publication. In other words, we gathered the citations of a paper published in 1999 for the period 2000 to 2004. Secondly, all articles and proceedings papers—both the cited and the citing—were categorized in six percentile rank classes (99^th^, 95^th^, 90^th^, 75^th^, 50^th^, and <50^th^). This normalization accords with that of the National Science Board of the U.S. National Science Foundation [22]: percentile rank classes are suited for identifying lowly-, medium- and highly-cited papers in a field. Both the National Science Board [23] and the Essential Science Indicators of Thomson Reuters classify papers as highly-cited if they belong to the top 1% of papers worldwide (that is, papers in or larger than the 99^th^ percentile).

The Ortega hypothesis predicts that highly-cited papers and medium-cited (or lowly-cited) papers would *equally* make references to papers with a medium impact (papers in the 50^th^ or 75^th^ percentile). The Newton hypothesis would be supported if the top-level research is more frequently based on previously highly-cited work (papers in the 99^th^ percentile) than that medium-level research cites highly-cited work. If scientific advancement is a result of chance processes (the Ecclesiastes hypothesis), no systematic association between the impact of cited and citing papers is expected.

## Results

**Figure 1:**
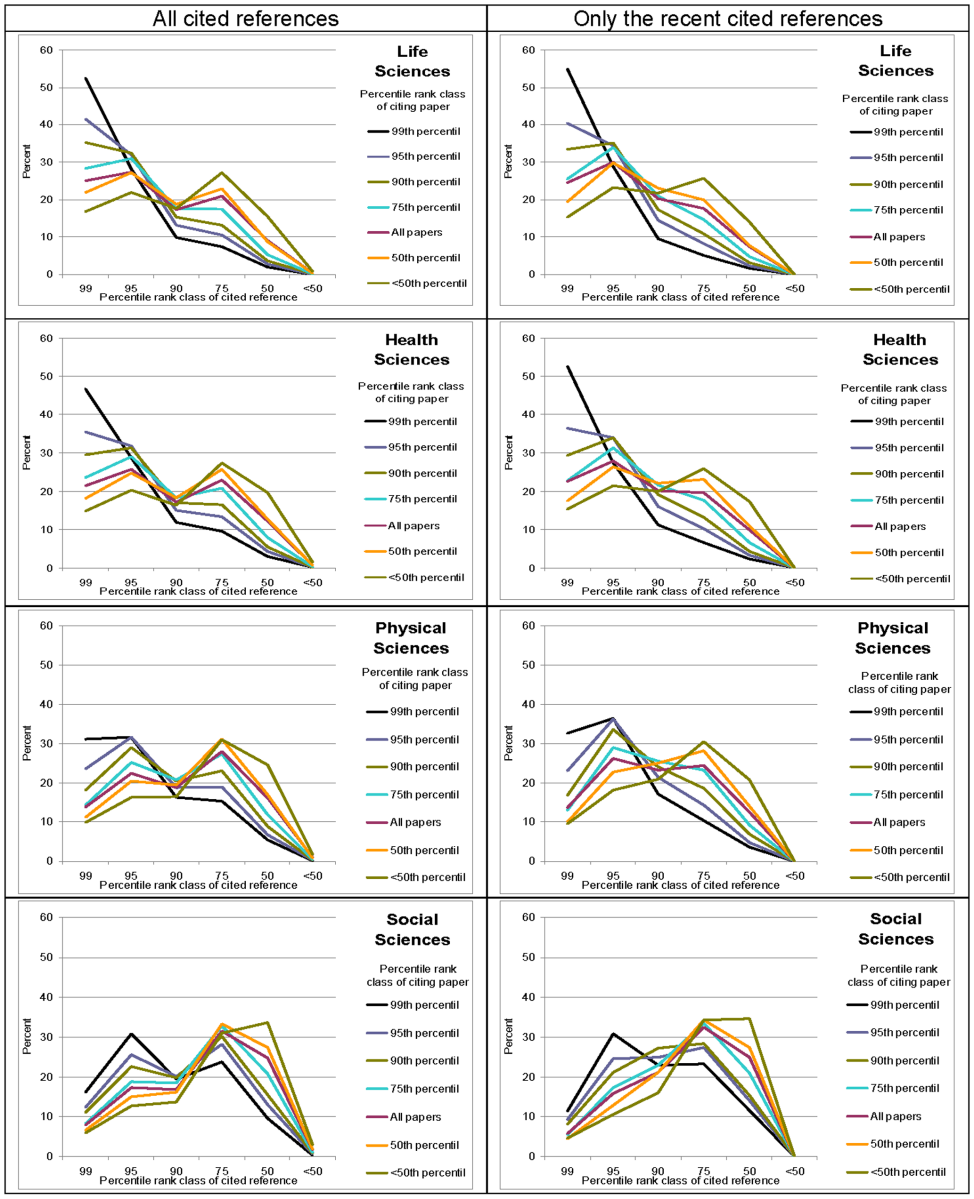
Share of cited references in papers published in 2003 in life sciences, health sciences, physical sciences and social sciences categorized into different percentile rank classes. The red lines refer to the cited references in all papers published in 2003; the other lines refer to cited references in papers with different citation impacts. The graphs in the left column are based on cited references from the years 1996 to 2003, and the graphs in the right column are based on cited references from 2002 only.

Figure 1 (left column) shows the percentile rank classes of the citing papers published in 2003 against the percentile rank classes of the cited references for each field. Both the ordinate and abscissa are used to describe the impact of the cited papers (cited references): The abscissa provides the percentile rank classes; the ordinate provides the percentage of the papers that belongs to this percentile rank class. The different impacts of the citing papers are shown by differently coloured lines. The share of cited references in the papers belonging to the 99^th^ percentile (i.e., the highly-cited papers in a field) is represented by a black line, 95^th^ by a purple line, 90^th^ by a green line, 75^th^ by a blue line, 50^th^ by an orange line, and <50^th^ by a brown line. The red line refers to the cited references in all citing papers published in 2003. Because citations to papers generally follow the well-known right-skewed distribution (many lowly or non-cited papers and only a few highly-cited papers) [24], the cited references in the figure are characterized by a citation impact that is at least at a medium level (50^th^ percentile or higher). In other words, the lower halfs of the distributions do not contribute to the citation patterns. As all cited references that are included in our study are cited at least once, the lowest impact class of cited references in all fields (all graphs in Figure 1) and of all percentile rank classes of citing papers (all lines in the graphs) is close to zero.

The graphs in Figure 1 (left column) show both similarities and differences among the four fields. In all fields, the high-impact papers (the 99^th^ percentile, that is, the black lines) cite high-impact papers to a larger extent than the papers in the other impact classes (e.g., the <50^th^ percentile, shown as brown lines). Conversely, medium-impact papers (50^th^ percentile or 75^th^ percentile, respectively, see the orange or blue line) cite medium-impact papers to a larger extent than high-impact papers (the 99^th^ percentile, the black line). This means for all four fields that (1) the high-impact research is connected to previously high-impact research more strongly than low- or medium-impact research, and (2) the lower the impact of a paper published in the four fields, the higher the share of cited references with medium impact. Both these findings support the Newton hypothesis for all four fields.

In addition to these similarities, there are also differences among the fields. First, in the life sciences and health sciences the differences among high-, medium-, and low-impact research in using preceding top-level research are large; in the physical sciences and especially in the social sciences these differences are much smaller. This could mean that the Newton hypothesis is valid to a different extent: our results suggest that this hypothesis is more corroborated in the life sciences and health sciences than in the physical sciences and social sciences. Second, the red lines in the four graphs of the figure (left column) which show the aggregate of papers in each field refer differently to highly-, medium-, and lowly-cited papers. Whereas in the life sciences and health sciences the share of cited references within the top-level impact class is larger than 20%, it is less than 20% in the physical sciences and less than 10% in the social sciences. Correspondingly, there is a high share of cited references in the case of the social sciences and physical sciences in the medium impact class; this share is significantly smaller in the life sciences and health sciences.

What are possible explanations for these differences among fields? The explanations could be technical or sociological in nature. A technical explanation might be that the differences in coverage of cited references by the Scopus database affect the results. Whereas in the life sciences 43% of the cited references were to journals indexed by Scopus, in the physical sciences, health sciences, and social sciences these percentages were 31%, 31%, and 3%, respectively. Perhaps, top-level research in the social sciences is predominantly published in media other than scholarly journals.

A sociological explanation could be that the results reflect differences in the paradigmatic fragmentation among the fields. Paradigms can be considered as clusters of theories and practices that determine the direction of research [25]. Publications in the life sciences are more codified across the board and one can also focus on commonly shared goals across disciplines more than in the social sciences [26]. The research traditions in several subfields grouped under the denominator of “social sciences” (e.g., economics, sociology) are organized in many schools of thought which are not strongly interconnected [27].

One would like to be able to control for whether the pattern in the data of Figure 1 (left column) could find its origin in “lazy authors citing the most obvious papers” rather than “giants citing other giants.” Perhaps, highly-cited papers were so often cited because of the “success-breeds-success” phenomenon [28], [29] in citation behaviour—rather than because of containing the crucial papers on which one builds—and thus overshadowing some innovative publications which would have deserved to be cited. In order to control for the validity of our results, therefore, we included in a second test only references from 2002 cited in papers published in 2003. One can assume that the authors of these papers could not know the subsequent citation impact of the papers published in 2002, since papers published in 2003 were written with only a few exceptions in 2002 or earlier.

This second analysis included (*i*) all articles and proceedings papers which were published in 2003 in the life sciences, health sciences, physical sciences, and social sciences (these are the same numbers as in the first analysis) and (*ii*) all articles and proceeding papers (published in 2002) which were cited within these publications: life sciences (n = 460,841 cited references), health sciences (n = 247,191), physical sciences (n = 428,305), and social sciences (n = 23,291). As in the first analysis, there are large differences in the numbers of cited references between the social sciences and the other three fields. The results of the second analysis are presented in the right column of Figure 1. The differences to the graphs displayed in the left column are small. The similarities of the results between the graphs in the left and right columns reveal that the authors apparently disregard the citation impact of the cited papers in their decisions to cite these papers.

## Discussion

In summary, highly-cited work in all scientific fields is more strongly based on previously highly-cited papers than on medium-cited work. Thus, we are able to demonstrate that papers contributing to the scientific progress in a field lean to a larger extent on previously important contributions than papers contributing little. In other words, the higher a paper's citation impact the stronger it is connected to preceding high-impact research (i.e., to research belonging to the 99^th^ percentile rank class). These findings support the Newton hypothesis and call into question the Ortega and Ecclesiastes hypotheses (given our usage of citation counts as a proxy for impact). Our results also suggest that medium-impact research plays a different role in the four fields: whereas in the social sciences and physical sciences scholars cite this underlying research, in the life sciences and health sciences the subtop is less important.

Given that research funding is commonly scarce, it is the responsibility of the scientific community to most effectively utilize the resources available.[30]. Our findings raise the issue of whether limited resources might best be concentrated in support of those scholars (research groups or institutions) who have already contributed to the literature by publishing high-impact papers (belonging to the 99^th^ percentile rank class). A concentration of resources on these elite structures seems to be practical especially for the life sciences and health sciences.

Indeed, current courses of action in research funding follow the concentration of scarce resources on outstanding researchers. The Wellcome Trust will allocate 20% of its total budget to an Investigator Awards program [31]. This program will fund only the very best scientists to investigate challenging and long-term research questions. The German Max Planck Society follows the so-called Harnack Principle. One formulates at the website as follows: “Max Planck Institutes are established only where the world's leading researchers are found. The researchers themselves determine their research topics and they are given optimum working conditions and the freedom to choose who they want to work with them” (see http://www.mpg.de/english/aboutTheSociety/missionStatement/excellencePrinciple/harnackPrinciple/index.html;accessed September 2010). The U.S. National Institutes of Health supports researchers with similar programs. Against the backdrop of our findings, these courses of action seem to be sensible especially in the life sciences and health sciences. In these fields, one can probably follow the argument of Cole and Cole [13] that the progress of science would be little impeded if only scientific excellence were supported. It should be tested in follow-up studies in the next years whether this statement can be hold.

Several limitations may have affected our results that should be considered in future studies: (1) It is not yet clear (especially for the social sciences) whether citation impact is a good approximation of actual research impact and of the role of research in scientific advancements [32], [33]. Furthermore, bibliometric research is limited to the analysis of scientific publications. The Ortega hypothesis relates explicitly to the experimental sciences thus including not only the previous literature as the basis of scientific progress but many different kinds of assistance (like technical support, sponsorship, and whatsoever). Newton [1], [2] refers to what he had done as a theoretician “on the shoulders of giants,” which is more likely to be covered by literature. (2) Our data indicate that a notable percentage of the papers cited in top-level papers is itself classified as medium-impact work. One does not know whether important contributions at the research front could have been made if only the top-level work had been available for referencing. (3) Although de Solla Price [34] has postulated an “immediacy factor” in which most scientific publications usually cite recent work, and papers tend to become obsolete within five to ten years [35], it might be that limiting our data to papers published after 1996 (that is, by using Scopus) affects the results [36]. If possible, this study should be replicated using the (Social) Science Citation Index which contains the historical backlog.

(4) The databases are restricted to mainly international journals and papers published in English. This restriction cannot be avoided by using current literature databases, but may affect especially the results for the social sciences. (5) It could be interesting to repeat the same analyses excluding self-citations. Although Boyack and Klavans [37] showed that self-citations cannot explain the strong association between citation impacts of the cited and citing papers at the aggregate level, the strong connection between current and previous top-level research in the life sciences and health sciences might partly be the result of large research programs that cite to a large extent internally. However, the systematic correction for self-citations is nearly impossible at the author level because of the strong homonymies among author names. For example, the Scopus database covered 8,173 documents of authors with the name “Singh” in 2009.

We proceed on the assumption that these limitations do not affect our results to such an extent that they are not valid.
